# Glycine therapy inhibits the progression of cataract in streptozotocin-induced diabetic rats

**Published:** 2012-02-11

**Authors:** Fereshteh Bahmani, S. Zahra Bathaie, S. Javid Aldavood, Arezou Ghahghaei

**Affiliations:** 1 Department of Clinical Biochemistry, Faculty of Medical Sciences, Tarbiat Modares University, Tehran, Iran; 2 Department of Clinical Sciences, Faculty of Veterinary Medicine, University of Tehran, Tehran, Iran; 3 Department of Biology, Faculty of Science, University of Sistan and Baluchestan, Zahedan, Iran

## Abstract

**Purpose:**

The purpose of this paper was to investigate the effect of the oral administration of L-glycine (Gly) on the development of diabetic cataract induced by streptozotocin (STZ) in rats.

**Methods:**

Two groups of male Wistar rats were intraperitoneally injected with a single dose of STZ (65 mg/kg bodyweight). Then, one group of diabetic rats and a control group were administered with 1% of Gly in drinking water for three months, ad libitum. Cataract development was monitored biweekly through ophthalmoscope inspection and was classified into four stages. At the end of 12 weeks, the animals were sacrificed and some biochemical parameters were determined in their lenses. The parameters include advanced glycation end products (AGEs), glycated proteins, total and soluble protein, glutathione (GSH), superoxide dismutase (SOD), catalase (CAT), aldose reductase (AR), and sorbitol dehydrogenase (SDH). Some parameters were also determined in the serum and blood of the rats.

**Results:**

Diabetic cataract gradually progressed in the STZ-administered group with no other treatment. Consequently, up to the end of the experiment, 2/3 of the animals in this group reached to the last stage of the cataract (mature cataract). The progress of this process was much slower in the diabetic group that was treated with Gly. At the end of the study, the visual cataract score was significantly lower in the diabetic group treated with Gly compared to those administered with STZ. Some lens parameters, including glycated proteins, AGEs, SOD, and AR activities, were increased while some others, including soluble and total protein, GSH level, and CAT activity, were decreased due to diabetes induction. After Gly treatment, all the above-named parameters had reverse changes except for the CAT activity. The SDH activity in the lenses had no changes due to diabetes or treatment. In addition, this treatment significantly decreased the amount of serum glucose (Glc), serum AGEs, and glycated hemoglobin (HbA1c) in the diabetic rats. Gly also increased the ferric reducing antioxidant power (FRAP) in the serum of diabetic rats. However, the decreased bodyweight of animals due to diabetes induction was not compensated by Gly administration. It is important to note that Gly had no effect on normal rat parameters.

**Conclusions:**

The results indicated that the oral administration of Gly significantly delayed the onset and the progression of diabetic cataract in rats. These effects were due to its antiglycating action and to a lesser extent, due to the inhibition of oxidative stress and polyol pathway.

## Introduction

Chronic hyperglycemia is a major determinant in the development of secondary complications of diabetes, such as diabetic cataract. Evidence indicate that both the duration of diabetes and the quality of glycemic control are the most important risk factors for cataract formation [1]. Cloudiness or opacification of the lens, which is responsible for focusing light and for producing clear and sharp images, is a characteristic feature of a cataract. Cataract is the leading cause of blindness across the world, especially in developing countries. Because of the high prevalence of diabetes in these countries, diabetic cataract may pose a major problem in the management of blindness [2,3]. Today, the only treatments for cataract are surgery and intraocular lens implantation, which are associated with significant cost and are not readily available for everyone. In addition, these treatments may give rise to serious complications, such as corneal edema, raised intraocular pressure, and so on. The complications may occur at the time of surgery or after that [4]; therefore, the identification of effective and non-toxic materials to prevent opacification of the lens is still important for diabetes research and pharmaceutical development.

The diffusion of extracellular glucose into the lens is not controlled by insulin. However, different mechanisms are involved in the development of cataract due to hyperglycemia [5]. Among the proposed mechanisms for the opacification of the lens, the posttranslational modification of the lens proteins and enzymes through reactions, such as non-enzymatic glycation, play a major role in cataractogenesis.

Non-enzymatic glycation involves the condensation reaction of the carbonyl group of sugar aldehydes with the NH_2_-terminus or free-amino groups of proteins. The initial product of this reaction is called a Schiff base, which spontaneously rearranges itself into an Amadori product. These compounds are relatively stable intermediates that can undergo a series of complex reactions that may lead to the formation of advanced glycation end products (AGEs) [6,7]. In some locations with limited protein turnover, such as in the lens fiber cells, protein glycation due to hyperglycemia may be increased up to tenfold [8]. Glycation causes the conformational change, aggregation, and cross-linking of proteins, which, in turn, leads to the formation of insoluble materials that are responsible for diabetic cataract [9]. Therefore, inhibition of this harmful process may prevent the progression of diabetic complications, including cataract.

A wide variety of anti-cataractogenic agents, such as anti-glycating compounds and AGE inhibitors, have been investigated in several in vitro and in vivo studies [10]. The role of amino acids in preventing diabetic complications was suggested [11]. The preliminary observations about the reduced glycation of lens proteins by glucose or galactose in the presence of certain amino acids are evidence [12,13]. In addition, the anti-cataractous effect of lysine and the mixture of some amino acids were shown in the animal model [14]. However, a comprehensive study about the anti-cataractogenic effect of glycine (Gly) is not seen in the literature based on our review.

Our previous works focused on preventing the effect of various chemicals, such as amino acids, on diabetic complications [11,15-18]. In the present study, we investigated the inhibitory effect of Gly on the progression of eye lens opacification in STZ-diabetic rats for a period of 12 weeks. At the end of the experiment, some biochemical parameters of the blood and lens of the animals were also determined and the effect of Gly on diabetic cataract was evaluated.

## Methods

### Materials

Streptozotocin (STZ), L-Glycine (Gly), Oxalic acid, 5-hydroxymethylfurfural (5-HMF), 2-thiobarbituric acid (TBA), Triethanolamine, nicotinamide adenine dinucleotide phosphate reduced form (NADPH), nicotinamide adenine dinucleotide reduced form (NADH), protein, and superoxide dismutase (SOD) quantification kits were purchased from Sigma Chemical Company (St. Louis, MO). All other chemicals and solvents were of analytical grade.

### Experimental design

The experiments were performed using male Wistar rats (7–8 weeks old and weighing 213±21.5 g). The rats were housed under a 12 h:12 h light-dark cycle at about 25 °C, room temperature. All animals had unlimited access to water. Animal care and protocols were in accordance with and approved by the Institutional Animals Ethics Committee of Tarbiat Modares University and conformed to the ARVO Statement for the Use of Animals in Ophthalmic and Vision Research.

After one week, 20 rats were randomly selected as the normal groups. Half of them were administered with Gly, as diabetic group under treatment. These groups were named as N and NG, respectively. Thirty-five of the rats were injected intraperitoneally with a single dose of streptozotocin (STZ; 65 mg/kg of bodyweight) [14]. They were divided into two groups with or without treatment with Gly and were named DG and D, respectively. STZ was dissolved in 0.1 M of sodium citrate buffer, with a pH of 4.5. It was kept on ice and was used within 10 min of dissolving. The control groups of rats (groups N and NG) were injected with the vehicle alone. Three days after STZ administration, only the rats that have a blood glucose level of >15 mmol/l were considered as diabetic and were included in our experiments.

The treatment of the diabetic and normal groups (groups DG and NG) with Gly (1% in drinking water) [19] was begun after one week (time zero in the figures and tables). The experiments lasted over 12 weeks.

### Blood measurement

Blood samples were collected from the orbit vein at the beginning, middle, and end of the study. EDTA-treated whole blood samples were saved for HbA1c determination and sera samples, prepared through 15-min centrifugation of blood at 5,000× g to separate clot, and stored at −70 °C for further studies.

Sera glucose was measured using the enzymatic colorimetric method (ELITech, SEES, France). For this, the Autoanalyser Model Biotecnica BT 3500 (Biotecnica, Rome, Italy) was employed. Glycated hemoglobin (HbA1c) in blood samples were measured with the use of the reagent kit and DS5 instrument of Drew Scientific Ltd. (Barrow-in-Furness, Cumbria, UK).

Determination of AGEs was performed according to the method of Kalousova et al. [20]. Blood serum was diluted 1:50 with PBS, pH 7.4. Flourescence intensity was recorded at the emission maximum (440 nm) upon excitation at 350 nm using the spectrofluorometer Shimadzu, Model RF 5000 (Shimadzu, Kyoto, Japan). Fluorescence intensity was expressed as a percentage of fluorescent emission (FI %).

The reducing ability of the biologic samples was determined using the ferric reducing antioxidant power (FRAP) assay of Benzie and Strain [21]. The FRAP assay measures the change in the absorbance of the FRAP reagent at 593 nm (Spectrophotometer Shimadzu, Model 3101) due to the formation of a blue-colored Fe II-tripyridyltriazine complex from colorless oxidized Fe III formed by the action of electron-donating antioxidants in the serum.

### Evaluation of cataract development

The progression of the cataract was monitored biweekly using a handheld ophthalmoscope that was equipped with a slit lamp by an individual who had no prior knowledge of the affiliation of the animal to the experimental group. Cataract formation was scored essentially according to the classification of Ao et al. [22] or Suryanarayana et al. [23] as follows: clear normal lens (O), peripheral vesicles (I), peripheral vesicles and cortical opacities (II), diffuse central opacities (III), and mature cataract (IV). Cataract formation was considered complete (grade IV) when the red fundus reflex was no longer visible through any part of the lens and when the lens appeared dull white to the naked eye.

### Lens preparation

At the end of 12 weeks, the animals were killed and their eyeballs were removed for biochemical evaluation. The eyeballs were soaked in 0.9% neutral normal saline and the lenses were dissected using the posterior approach then placed into pre-weighed Eppendrof tubes and frozen at −70 °C until further analysis. A 10% homogenate was prepared from the lens in 50 mM phosphate buffer (pH 7.4). The activity of the lens enzymes and soluble protein were measured in the soluble fraction of the lens homogenate (15,000× g at 4 °C) while the lens AGEs and the total protein were determined in the total homogenate.

### Biochemical assays

The determination of the lens AGEs was performed as described by Ranjan et al. [24]. Fluorescent measurements were performed in the total lens homogenate samples using a Spectrofluorometer Shimadzu, Model RF 5000 and the fluorescence intensity was recorded at the emission maximum (440 nm) upon excitation at 366 nm. Results were expressed as a percentage of fluorescent emission (FI %).

Determination of the amount of glycated lens proteins was based on the method described by Blakytny and Harding [25]. The 5-hydroxymethylfurfural (5-HMF) was released into the solution due to the boiling of the glycated protein in the presence of a weak acid. Any solubilized protein was then precipitated out of the solution through centrifugation. After the addition of thiobarbituric acid (TBA), it forms adduct with 5-HMF that absorbs at 443 nm. The amount of glycation was determined using the standard graph that was drowned by various concentrations of 5-HMF.

Measurement of free, reduced GSH in the lens was performed using the 5,5′-dithiobis-[2-nitrobenzoic acid] (DTNB) method [26]. The lens was ground in 1 ml of 10% trichloroacetic acid (TCA) then left for 20 h before centrifugation at 11,500× g at room temperature for 20 min. The supernatant was removed into a tube and the precipitate was washed twice with 10% TCA. Then, the supernatants were combined; 0.5 ml of supernatant from each sample was made up to 1 ml by adding 10% TCA and 2 ml of 1 M Tris, pH 9.0 followed by the addition of 50 μl DTNB (dissolved in ethanol at a concentration of 3.965 mg/ml) and mixed well. Standards were made using different volumes of 0.1 mM reduced GSH in 10% TCA and making up to 1 ml with 10% TCA. Reaction blanks consisted of 1.0 ml of 10% TCA, Tris, and DTNB. The reaction solutions stood for 5 min at room temperature and the absorbance was read at 412 nm. The concentration of GSH was read from a standard graph using GSH.

Lens superoxide dismutase (SOD) levels were determined using the method of Sun et al. [27], based on the inhibition of nitroblue tetrazolium. In the assay, the xanthine-xanthine oxidase system was used as a superoxide generator. The absorbance of the reduction product (formazane dye) was measured at 440 nm. The SOD activity was determined by the degree of inhibition of this reaction. The catalase (CAT) activity in the lens was assayed with hydrogen peroxide as the substrate using a method that was based on the direct measurement of H_2_O_2_ decomposition [28].

Aldose reductase (AR) and sorbitol dehydrogenase (SDH) activities were estimated according to the methods described by Kinoshita et al. [29] and Gerlach and Hiby [30], respectively.

The total and soluble proteins of the lens were measured using the method of Lowry and by using a protein assay kit from Sigma Company.

### Statistical analysis

Data was expressed as mean±SD. One-way ANOVA was used for testing the statistical significance between groups. Statistical analysis of the average of the cataract score of the lens opacity was done using the Mann–Whitney U-test. A p<0.05 was considered significant. All data was dealt with using the SPSS 16.0 statistical package (IBM, New York, NY).

## Results

### General parameters

All animals in the normal groups (treated or untreated with exogenous Gly) lived up to the end of the experiment. Out of the 35 STZ-treated rats, 32 contracted diabetes. Mortality in the experimental groups after five and nine weeks was five (5 of 17) rats in the diabetic groups and two (2 of 15) rats in the treated group administered with Gly, respectively. Thus, 70.6% and 86.7% of the rats in these groups and all of the animals in the control groups (with or without treatment by Gly) remained up to the end of the experiment.

**Figure 1:**
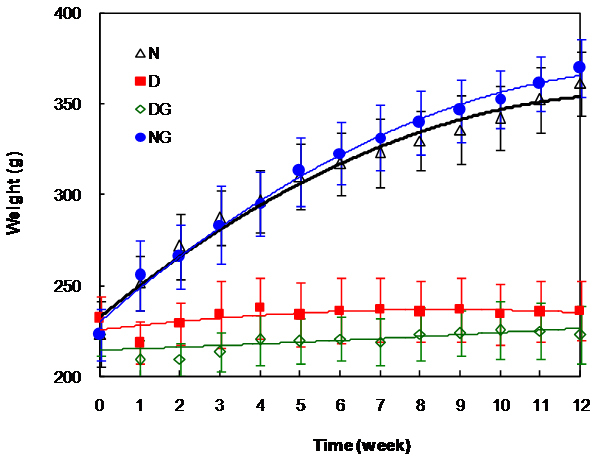
Changes in the rat’s bodyweight during the course of the experiment. The data shows the average results in all the animals in a given group at a given time. The weights of the normal groups were significantly different from those of the diabetic groups (p<0.01) and there were no significant differences between the two diabetic groups at any time. N: normal rats; D: diabetic rats; DG: diabetic rats with Gly; and NG: normal rats with Gly.

Table 1 shows the changes in the bodyweights of all four groups of animals during the course of this study. The bodyweights of the two diabetic groups were significantly (p<0.01) lower than those of the normal groups and Gly had no effect on this parameter (Figure 1).

**Table 1:** The effect of Gly on the bodyweights of all groups of rats during the experiment.

** **	**Time (week)**												
**Group name**	**0**	**1**	**2**	**3**	**4**	**5**	**6**	**7**	**8**	**9**	**10**	**11**	**12**
N	223.3 ±18.1 (n=10)	251.6 ±15.1 (n=10)	271.6 ±18.3 (n=10)	287.5 ±15.1 (n=10)	296.6 ±17.2 (n=10)	310.0 ±18.2 (n=10)	317.5 ±17.2 (n=10)	323.3 ±18.8 (n=10)	330.0 ±16.7 (n=10)	335.8 ±18.8 (n=10)	342.5 ±17.5 (n=10)	352.5 ±17.8 (n=10)	361.6 ±17.8 (n=10)
D	232.2 ±11.5 (n=17)	218.3 ±11.7 (n=17)	228.8 ±11.7 (n=17)	234.4 ±18.4 (n=17)	237.7 ±16.8 (n=17)	233.8 ±17.5 (n=17)	236.1 ±18,0 (n=15)	236.6 ±17.7 (n=15)	235.5 ±16.9 (n=14)	236.6 ±17.3 (n=13)	234.4 ±16.7 (n=13)	235.5 ±16.9 (n=12)	236.1 ±16.5 (n=12)
DG	222.7 ±11.8 (n=15)	208.8 ±11.4 (n=15)	209.4 ±9.2 (n=15)	213.3 ±10.9 (n=15)	220.0 ±13.5 (n=15)	219.4 ±12.1 (n=15)	220.5 ±12.1 (n=15)	218.8 ±12.7 (n=15)	223.3 ±14.8 (n=15)	223.8 ±12.4 (n=15)	225.5 ±16.1 (n=14)	225.0 ±15.2 (n=14)	222.7 ±16.0 (n=13)
NG	223.0 ±14.0 (n=10)	255.5 ±19.4 (n=10)	266.0 ±17.3 (n=10)	283.5 ±21.6 (n=10)	295.5 ±17.6 (n=10)	313.0 ±18.9 (n=10)	323.0 ±17.0 (n=10)	331.5 ±18.0 (n=10)	340.0 ±17.3 (n=10)	346.5 ±17.5 (n=10)	353.0 ±15.8 (n=10)	361.5 ±15.3 (n=10)	370.0 ±16.2 (n=10)

The data are mean±SD. There was a statistically significant difference between the diabetic rats and normal rats (groups D and DG versus N, p<0.01) from one week to the end of the experiment. The bodyweights of the rats in the normal group receiving Gly were not different from the normal group that did not receive Gly. N: normal rats; D: diabetic rats; DG: diabetic rats with Gly; and NG: normal rats with Gly.

### Biochemical parameters in the serum or blood

**Figure 2:**
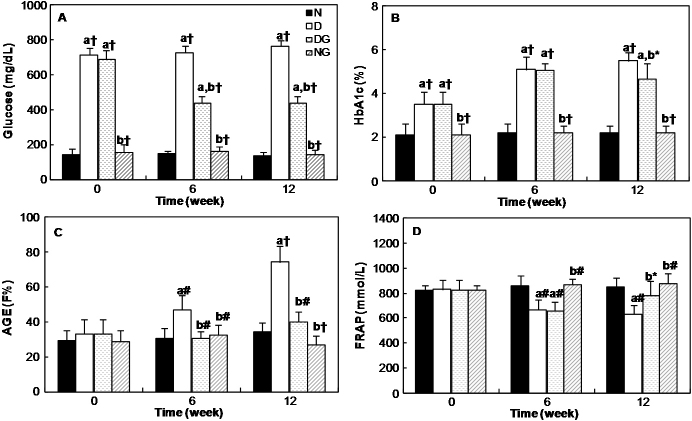
Changes in the serum and blood biochemical parameters in different groups of rats at the different times of the experiment. **A**: Glucose concentration, **B**: Percent of HbA1c, **C**: Fluorescence intensity of AGEs, and **D**: FRAP. N: normal rats; D: diabetic rats; DG: diabetic rats with Gly; and NG: normal rats with Gly. “a” indicates the significance of the data that compares group N versus all groups. “b” indicates the significance of the data that compares group D versus groups of glycine-treated rats. *, p<0.05; #, p<0.01; and †, p<0.001.

Figure 2A shows the changes in the serum glucose level of all four groups of rats during the course of the experiment. The glucose level in the sera of both diabetic groups are higher than the normal group, but after the administration of Gly, the glucose concentration reduced significantly (p<0.001) in the diabetic group.

As seen in Figure 2B, the diabetic rats had a higher blood HbA1c level than the normal rats throughout the experimental period. The percentage of HbA1c showed no significant differences among diabetic rats until six weeks, but after 12 weeks, a significant (p<0.05) decrease in the mean percent of HbA1c in diabetic rats treated with Gly in comparison with the diabetic rats without treatment was observed.

The formation of the AGEs in the serum of the diabetic groups increased gradually. However, it was decreased and closed to the normal value in the diabetic group that was under treatment with Gly. Significant differences were observed in this parameter between the diabetic group and the other three groups (p<0.01; Figure 2C).

The results of the FRAP assay, as a measure of the antioxidant activity, in the serum of rats are shown in Figure 2D. It indicates that this parameter decreased significantly in the diabetic group, but after 12 weeks of treatment with Gly, it increased significantly (p<0.05).

### Cataract formation

**Figure 3:**
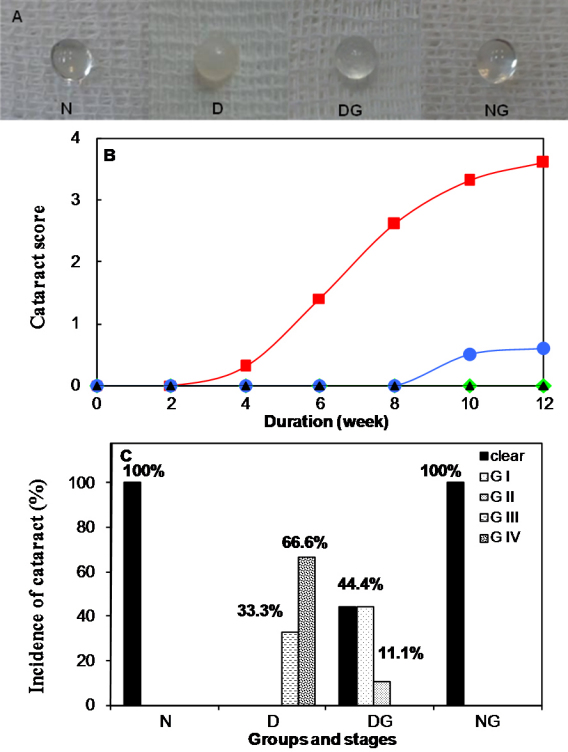
Effect of Gly therapy on the opacity of the lenses in STZ-diabetic rats. **A**: Photographs of lenses from each group at the end of 12 weeks. **B**: Progression of cataract in diabetic rats throughout the experimental period. Cataract formation was scored biweekly according to the following classification: clear normal lens (O), peripheral vesicles (I), peripheral vesicles and cortical opacities (II), diffuse central opacities (III) and mature cataract (IV). The scores of cataracts in each group were averaged at the given time and the average score of the cataract was plotted as a function of time. There was a significant difference (p<0.001) between the average score of the cataract of groups D and DG from six weeks to the end of the study. **C**: Maturation of the cataract in diabetic rats after 12 weeks. Cataract development in rats was observed on week 12 of the study and the number of lenses that developed opacity against the total number of lenses was considered for calculating the percentage of incidence of cataract in each group. N: normal rats; D: diabetic rats; DG: diabetic rats with Gly; and NG: normal rats with Gly.

Figure 3A shows the representative photographs of the lenses from each group at the end of the study. The onset of cataract in the diabetic group was observed five weeks after STZ injection (Figure 3B). In the diabetic group, the rats developed cataract more or less at the same rate. After eight weeks, 33.3% of the lenses of the untreated diabetic rats were in grade II, 66.6% in grade III and none of them were clear. Conversely, at this time, all the lenses in the diabetic group that was treated with Gly were clear. On the 10th week, 33.3% of the lenses of the animals in group D developed mature cataracts (grade IV) and 66.6% were in grade III. The treatment of diabetic rats with Gly delayed the onset of the cataract because 55.6% of the lenses of this group were in grade I and 44.4% were clear at the end of the 10^th^ week. At the end of the experiment (week 12, Figure 3C), all the lenses of the animals in the untreated diabetic group showed an advanced stage of cataract (grade III and IV). On the other hand, in Gly-treated diabetic rats, 44.4% of the lenses remained clear, 44.4% were in grade I and only 11.1% were in grade II. All the lenses in the normal groups (group N and NG) remained clear throughout the experimental period.

### Biochemical parameters of the lens

There was an increase of AGE fluorescence in the lenses of the diabetic group in comparison with the normal one. Gly significantly (p<0.001, Table 2) decreased this parameter in diabetic rats.

**Table 2:** The effect of glycine on the AGE fluorescence emission, glycated protein, and protein content of rat lenses.

**Group name**	**Lens AGE (FI %)**	**Glycated proteins (μmol of HMF/mg pr)**	**Total protein (mg/lens)**	**Soluble protein (mg/lens)**
N	18.5±3.1	0.52±0.06	17.68±0.72	14.50±0.66
D	82.6±5.9^a†^	1.87±0.30^a†^	10.19±3.10^a#^	4.79±2.28^a#^
DG	23.7±4.9^b†^	0.86±0.15^b†^	16.69±2.02^b#^	8.25±0.84^a,b#^
NG	19.3±3.7^b†^	0.50±0.14^b†^	17.17±1.47^b#^	13.97±1.04^b#^

Data are expressed as the mean±SD (n=6). “a” indicates the significance of the data that compares group N versus all groups. “b” indicates the significance of the data that compares group D versus groups of Glycine-treated rats. #, p<0.01; and †, p<0.001.

Hyperglycemia causes an increase in the glycated protein of the diabetic lenses. Significant differences in the glycated protein between the normal group (group N) and the diabetic group (group D) were noted. Gly administration had a significant (p<0.001; Table 2) effect on the decrease of the formation of glycated protein in the diabetic rats that were under treatment.

The total and soluble protein content in the lens of all four groups was also determined. There was a significant decrease of both total and soluble protein levels in diabetic rats compared to the normal rats (Table 2). Treatment with Gly caused a significant (p<0.01) increase in the protein levels of diabetic lenses.

The amount of glutathione (GSH), which acted as a reducing peptide, and CAT, which acted as an antioxidant enzyme, decreased in the lenses of untreated diabetic rats 13 weeks after STZ injection when they were compared with the normal group (Table 3). Gly had a statistically significant (p<0.05) effect in raising the amount of GSH in the diabetic group that was under treatment. No significant changes were observed in the CAT activity (Table 3). On the other hand, the activity of the SOD increased in the lenses of the diabetic group (Table 3) and significantly (p<0.05) decreased after the treatment of this group with Gly.

**Table 3:** The effect of glycine on the content of glutathione and the activities of antioxidant enzymes in rat lenses.

**Group name**	**GSH (mg/g lens)**	**CAT activity (μmol/g lens pr)**	**SOD activity (units/min/mg lens pr)**
N	178.0±36.9	59.8±8.6	6.20±0.20
D	51.0±14.8^a†^	31.7±11.5^a#^	15.39±5.34^a†^
DG	126.9±25.5^b*^	39.1±10.7	11.09±1.72^a,b*^
NG	175.0±23.6^b†^	55.7±13.2^b*^	6.50±0.45^b†^

Data are expressed as the mean±SD (n=6). “a” indicates the significance of the data that compares group N versu all groups. “b” indicates the significance of the data that compares group D versus groups of Glycine-treated rats. *, p<0.05; #, p<0.01; and †, p<0.001.

AR is the key enzyme of the polyol pathway and its activity was significantly higher in the diabetic group than in the normal one. Meanwhile, the activity of the other enzyme of this pathway, SDH, was not significantly altered (Table 4). The treatment with Gly caused a significant (p<0.05) decrease in the AR activity of diabetic rats, but had no effect on the SDH activity.

**Table 4:** The effect of glycine on the activities of the polyol pathway enzymes in rat lenses.

**Group name**	**AR activity (μmol NADPH oxidized/hr/100 mg pr)**	**SDH activity (μmol NADH oxidized/hr/100 mg pr)**
N	30.8±3.1	4.63±0.54
D	40.2±3.0^a†^	5.12±0.84
DG	32.8±2.3^b*^	5.14±0.59
NG	29.9±4.9^b†^	4.58±0.90

Data are expressed as the mean±SD (n=6). “a” indicates the significance of the data that compares group N versus all groups. “b” indicates the significance of the data that compares group D versus groups of Glycine-treated rats. *, p<0.05; and †, p<0.001.

## Discussion

Cataract is an opacity within the lens that interferes with vision. It is the most frequent cause of visual impairment throughout the world. This condition usually happens as a consequence of diabetes and hyperglycemia [5]. Hyperglycemia may be controlled in different ways, such as dietary changes, the use of hypoglycemic agents, insulin, and through islet transplantation. Cataract, as a long-term complication of diabetes, has remained a serious problem. At present, the only treatment for cataract is surgery. If there is a possibility that the onset of cataract can be delayed by 10 years, the need for cataract surgery may be decreased by as much as half [31]. Therefore, any strategy that prevents or slows down the progression of cataract can have a significant impact on human health.

There are possible mechanisms involved in the pathophysiology of diabetic cataract. These include increased formation of advanced glycation end products (AGEs), oxidative stress, and increased polyol pathway or osmotic stress [5]. Although there is cross talk between these pathways, results of various studies suggest that glycation and oxidative stress are two of the major determinants in the pathogenesis of diabetic cataract [24,32].

It was previously shown that amino acids may be effective in the prevention of diabetes and its complications [33]. It has also been shown that Gly has antioxidant properties and that it inhibits non-enzymatic glycation [19,34-36]. Therefore, as a continuation and follow up to our recent investigation on the therapeutic and preventative effects of some chemicals [11,16-18] and natural products [37] on diabetic complication, we used Gly to treat the STZ-induced diabetic rats and investigated its effect on the prevention of cataract in our present study.

As the results indicate, there were no differences between the determined parameters in the normal rats (N) and in the normal rats treated with Gly (NG). Therefore, Gly has no toxic effect on healthy rats and it even decreased the mortality of diabetic animals. The safe dose for Gly administration has been reported previously by others [19,36]. In addition, our data indicate the significant reduction of the serum Glc level of diabetic rats after six weeks of treatment with Gly. A similar effect was observed on the diabetic rats that were orally administered with Gly [19]. However, the specific mechanism of Gly- induced diminution of blood Glc is still not known, but the reaction of Gly with sugars, such as Glc has been observed. This means that the non-enzymatic glycation of Gly decreases the free Glc concentration in the medium [34]. In addition, in humans, it has been found that glycine administration increases the response to insulin [38]. Oral glycine stimulates the secretion of a gut hormone that potentiates the effect of insulin on glucose removal from the circulation [39]. However, the serum glucose level of diabetic rats treated with Gly is still high and is more than the threshold (180 mg/dl) that is necessary for the induction of cataract [40]. Our results also indicate that cataract was formed in the diabetic group after the fifth week of STZ injection and progressed up to the end of study. However, the significant decrease in the cataract score is seen due to the Gly administration. Therefore, the observed delay of the onset and inhibition of the progression of cataract after the administration of Gly in diabetic rats is possibly due to other factors in addition to its Glc lowering property. To investigate this hypothesis, various biochemical pathways related to the formation of diabetic cataract were investigated.

A significant decrease was observed in the HbA1c and AGEs of the diabetic group that was under treatment with Gly. Similar results have been reported after six-month treatment of diabetic rats with Gly [19,35]. We have also demonstrated a significant decrease in HbA1c and AGEs after L-Lys administration to the diabetic rats [11]. Gly administration also significantly increased the FRAP in the serum of the diabetic rats. A similar increase due to the administration of Lys, aspirin, and spermine in diabetic rats have also been shown by us in our previous studies [11,16,17]. The decreased levels of HbA1c and AGEs in diabetic rats treated with the above-named compounds, including Gly, should be explained by their inhibitory effect on protein glycation [11,16,17,34], thereby reducing the oxidative stress that is usually associated with increased FRAP.

During the progression of the cataract, the total and soluble protein content of the diabetic lenses was reduced significantly. The reduction may be due to the formation of high molecular weight aggregates of proteins, which may have resulted from the occurrence of oxidative stress and the cross link formation between protein free-SH groups, AGE formation, and protein leakage after the increased activity of the polyol pathway and from osmotic stress, which increased membrane permeability [9,31]. As our results show, the treatment of rats with Gly reduced protein glycation and AGEs formation. It also significantly prevented the total and soluble protein loss in diabetic lenses. The preventative effect of glycine on the lens proteins glycation in the glucose-treated homogenates of normal lenses was also shown previously [34]. The suggested mechanism for this action of Gly, as explained above, is its ability to bind excess Glc and reduce its free concentration [34].

The decreased GSH and the altered activities of the antioxidant enzymes, including CAT, are due to the increase of oxidative stress in diabetic conditions. Such alteration was also reported previously in various types of cataract, including diabetic cataract [32]. A significant increase in the activity of SOD in the lenses of diabetic rats was also reported previously [41]. Gly, with its antiglycating and antioxidant activities, could significantly increase the amount of GSH and decrease the activity of SOD enzyme in diabetic rat lenses. In several studies, the beneficial effects of antioxidants, such as curcumin, turmeric, N-acetylcysteine, and glutathione ethyl ester, have been shown on the prevention of diabetic cataract [31,32].

The dicarbonyl intermediates of the Maillard reaction, such as glyoxal and methylglyoxal, as the substrates of AR, are produced during the glycation process. When these compounds increase due to hyperglycemia, the activity of the polyol pathway is also increased [42]. AGEs are also inducers of AR and their increase during aging and diabetes are another reason for the enhancement of the activity of the polyol pathway [43]. As our results indicate, the activity of the AR was significantly increased due to diabetes induction and the treatment of diabetic rats with Gly caused a significant decrease in the formation of AGEs and the activity of this enzyme. However, SDH activity was not significantly altered in the diabetic group. A similar observation has also been reported previously by Saraswat et al. [31]. They reported that Zingiber officinalis have antiglycating property and that it can reduce non-enzymatic glycation and AGEs formation in diabetic rat lenses.

Finally, the beneficial effects of Gly on diabetic cataract may be summarized as follows:

1. High solubility in comparison with some other anti-cataract agents [36].
2. Hypoglycemic, antiglycating, and antioxidant properties.
3. Having specific transporter in the cortex and nuclear of lenses [44,45].
4. Physiologically available and nontoxic for non-neuronal tissues [44].

In conclusion, the present study shows that the administration of Gly to diabetic rats delays the onset and the progression of cataract by decreasing protein glycation, preserving protein content, potentiating the antioxidant system, and decreasing the activity of the polyol pathway in the lenses. Decreasing the serum Glc, AGE formation and HbA1c, in addition to the increase in the FRAP are also other reasons for the beneficial effect of Gly on diabetic cataract. Gly did not show any toxic effect on the above-named parameters in the normal rats.
